# Iron-Doped Molybdenum Sulfide Nanoflowers on Graphene for High-Performance Supercapacitors

**DOI:** 10.3390/molecules30204045

**Published:** 2025-10-10

**Authors:** Xuyang Li, Mingjian Zhao, Shuyi Li, Shiyuan Cheng, Yiting Zuo, Kaixuan Wang, Meng Guo

**Affiliations:** 1 School of Chemistry and Pharmaceutical Engineering, Nanyang Normal University, Nanyang 473061, China; lsy1259058642@163.com (S.L.); cyi2537653658723@163.com (S.C.); 13462297511@163.com (Y.Z.); 2 School of Biological and Chemical Engineering, Nanyang Institute of Technology, Nanyang 473004, China; 15333887635@163.com (M.Z.); eryang0924@163.com (K.W.)

**Keywords:** supercapacitors, power density, specific capacitance, cycling stability

## Abstract

Supercapacitors (SCs) are widely acknowledged for their high-power density as energy storage devices; designing electrode materials with both high efficiency and exceptional energy density remains a significant challenge. In this study, a flower-like iron-doped molybdenum sulfide on graphene nanosheets (FMS/G) was synthesized through a simple, efficient, and scalable solvothermal approach. The FMS/G composite demonstrated exceptional performance when employed as both positive and negative electrodes, owing to the effective incorporation of iron into the MoS_2_ crystal lattice. This doping induces defects and facilitates abundant redox reactions, ultimately boosting electrochemical performance. The FMS/G composite demonstrates an ultrahigh specific capacitance of 931 F g^−1^ at 1 A g^−1^, along with excellent rate capability, retaining 582 F g^−1^ at 20 A g^−1^. It also exhibits remarkable cycling stability, maintaining 90.5% of its initial capacitance after 10,000 cycles. Furthermore, the assembled FMS/G-3//FMS/G-3 supercapacitor device achieves a superior energy density of 64.7 Wh kg^−1^ at a power density of 0.8 kW kg^−1^ with outstanding cycling stability, retaining 92% of its capacitance after 10,000 cycles. The remarkable capabilities of the flower-like FMS/G composite underscore its noteworthy potential for promoting effective energy storage systems.

## 1. Introduction

Rising environmental pollution and the depletion of fossil fuel resources have increased the need for developing alternative, sustainable energy conversion and storage technologies. These devices should offer high energy with enhanced power densities while being cost-effective and environmentally friendly [1,2,3]. Supercapacitors (SCs), among the various energy storage options, have garnered significant attention in modern electronics because of their rapid charge–discharge capabilities, outstanding power density, and extremely long cycle life, setting them apart from traditional batteries [4,5,6]. However, the inherently low SC’s energy density limits their large-scale industrial application. Enhancing energy density requires advanced electrode materials with the capability to store a greater number of charge carriers, thereby improving the overall capacity. Consequently, exploring novel electrode materials that possess desirable characteristics, such as expansive surface area, high electrical conductivity, distinctive porous structure, along with ample void space, is essential for enhancing the energy density of supercapacitors without compromising power density as well as cycling stability. Furthermore, assembling SCs by connecting electrodes of different types (e.g., a capacitor and battery) can expand the operating potential window, leading to an improved energy density tailored for specific applications [7,8]. Unique and appropriate architecture bearing electrode material contributes significantly to the improvement of SCs’ electrochemical energy storage performance.

Recently, transition metal dichalcogenides (TMDs) have gained widespread attention for SC applications, building upon earlier research on transition metal oxides [9,10]. TMDs are generally represented by the formula MX_2_, where M denotes a transition metal and X corresponds to a chalcogen. In this structure, metal atoms contribute four electrons to fully occupy the bonding states of MX_2_, leading to oxidation states of +4 and −2 [11,12]. Especially when compared to a multitude of nanostructures, two-dimensional materials stand out as the most effective electrode materials for energy storage applications. Their unique planar structure endows them with a remarkably high specific surface area, which in turn facilitates extensive ion intercalation and enables a pseudo-capacitive mechanism for efficient energy storage [13]. The integration of MoS_2_ with graphene has demonstrated superior electrical properties and versatile applications, particularly excelling in energy storage systems [14,15]. MoS_2_ stores faradaic charges through two distinct mechanisms: interlayer and intralayer processes. Significant research attention has been devoted to the 1T/2H phase of MoS_2_, which exhibits remarkable SC characteristics. However, MoS_2_ faces some limitations, such as low-energy density and limited electrical conductivity [16]. To address these drawbacks, doping has proven effective in mitigating restacking and enhancing electrical conductivity, ultimately boosting the electrochemical performance of MoS_2_.

Doping with various transition metal ions stands out as a highly effective strategy for enhancing their electrochemical performance in energy storage applications [17,18]. This is because introducing dopants into the crystal lattice can create defects that occupy specific sites within the crystalline structure, thereby providing additional active regions to boost the material’s electrochemical behavior [19,20]. As electrical conductivity improves, the bandgap narrows, leading to a reduction in internal resistance. Simultaneously, the generation of more reaction sites increases the number of electrochemically active sites. This effectively modifies the electrical structure of the material, facilitating faster electron and ion transfer rates. Dopants such as Fe, Co, Cu, and Ni possess ionic radii similar to that of Mo in MoS_2_, enabling them to enhance its electrochemical properties. In this context, incorporating iron (Fe) into the MoS_2_ structure, with its distinctive nanostructure, is anticipated to enhance oxidation states and generate additional active sites, demonstrating enhanced electrochemical performance of SCs [21]. However, Fe-doped MoS_2_ (FMS) often experiences degradation in KOH electrolytes during prolonged charge–discharge cycles, leading to a diminished cycling life of the electrode. Moreover, the electrochemical performance of FMS tends to be unstable due to agglomeration [22]. To overcome these challenges, integrating FMS with a conductive substrate is crucial, as this strategy not only suppresses aggregation and degradation of active FMS but also facilitates electron transport, thereby ensuring enhanced cycling stability and long-term performance.

Graphene, among the wide range of conductive supports, has attracted considerable attention as an outstanding substrate because of its exceptionally high surface area, excellent electrical conductivity, and superior cycling stability [23,24]. Additionally, graphene enhances the nucleation and formation processes of MoS_2_. Consequently, FMS can be readily synthesized using a straightforward solvothermal method in the presence of graphene. In this scenario, graphene serves as an ideal substrate to mitigate the agglomeration of FMS, thereby improving its capacitive performance and ensuring excellent durability.

**Scheme 1:**
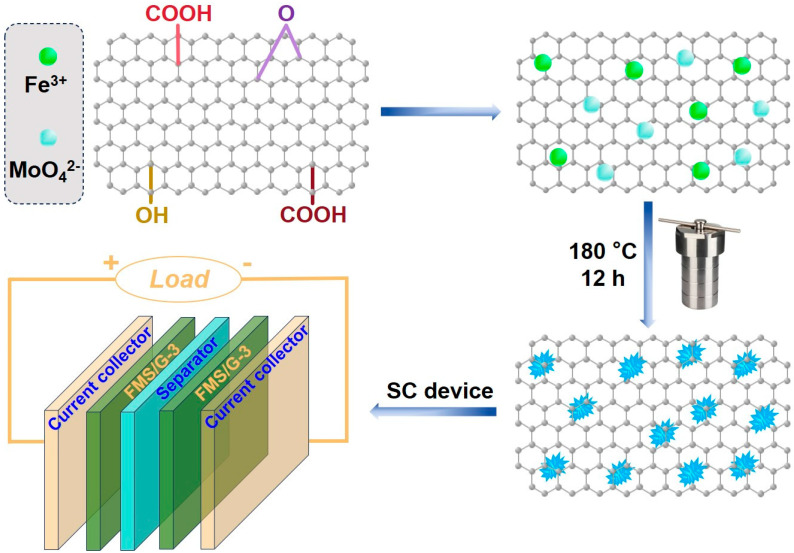
Depicts the preparation of FMS/G composite, achieved via a simple and efficient hydrothermal approach.

Herein, we report the development of a novel flower-like Fe-doped molybdenum sulfide anchored on graphene (FMS/G) electrode material (Scheme 1). The FMS/G composite is synthesized using a straightforward, scalable, and cost-effective solvothermal approach as shown in Scheme 1. In the resulting nanostructure, iron atoms serve as dopants within the MoS_2_ nanostructure, elevating the oxidation states and thereby enhancing electrochemical performance. Additionally, iron doping effectively mitigates the degradation of FMS nanoflowers (NFs) during prolonged charge–discharge cycles. The FMS/G composite delivered a high specific capacitance of 931 F g^−1^ at a current density of 1 A g^−1^, along with sustained long-term durability, maintaining 90.5% of its initial capacitance after 10,000 cycles. The outstanding electrochemical performance is ascribed to the incorporation of an iron dopant, which promotes faster charge transfer kinetics in the active electrode material. Furthermore, incorporating graphene as a support accelerates charge transfer processes and greatly strengthens the electrical conductivity of the composite. A supercapacitor device fabricated using FMS/G as both the positive electrode and negative electrode exhibits a superior energy density of 64.7 Wh kg^−1^ at a power density of 0.8 kW kg^−1^, coupled with outstanding cycling stability (92% capacitance retention after 10,000 cycles).

## 2. Results and Discussion

**Figure 1:**
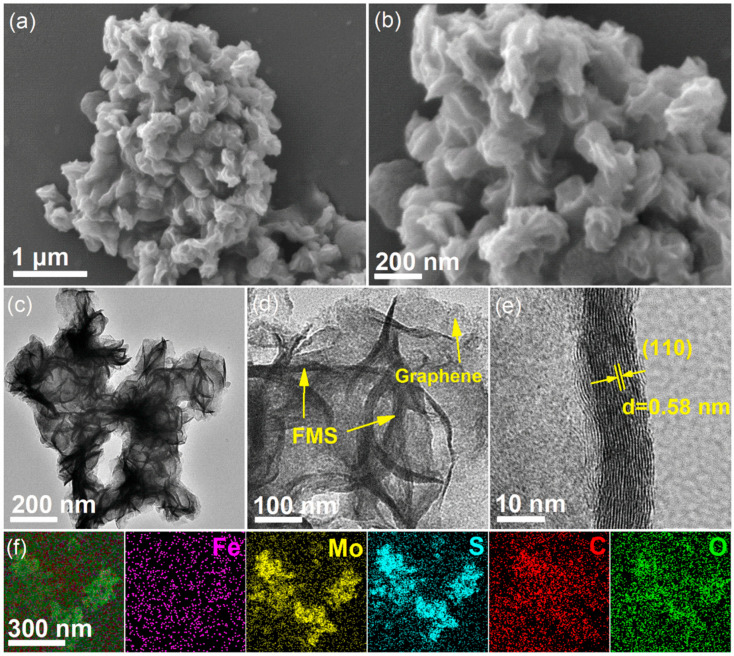
(**a**,**b**) SEM images, (**c**,**d**) TEM images, (**e**) HR-TEM image, and (**f**) EDS elemental mapping of Fe, Mo, S, C, and O of FMS/G-3 composite.

Figure 1a,b presents representative SEM images of the FMS/G-3 composite, revealing that the flower-like FMS/G nanostructures were evenly distributed on the graphene nanosheets, effectively preventing the agglomeration of the FMS nanoflowers. To further investigate the morphology, TEM as well as high-resolution TEM (HR-TEM) analyses were carried out. As depicted in Figure 1c, TEM images revealed that FMS nanoflowers were uniformly distributed over the graphene substrate. Figure 1d shows that each nanoflower consisted of nanosheets encapsulated by graphene layers. The HR-TEM image in Figure 1e displays a 0.58 nm interplanar spacing, corresponding to the (110) plane of MoS_2_ (JCPDS 74-0932) [25]. Furthermore, the active FMS nanoflowers are tightly wrapped by graphene nanosheets, highlighting the strong synergistic effect between the graphene support and FMS active sites [26,27]. The hydroxyl groups on graphene promote a strong synergistic effect that facilitates faster ion transfer between the electrolyte and electrode, resulting in superior capacitive behavior and greater electrochemical stability of the electrode material [28]. The scanning EDS mapping in Figure 1f shows that iron atoms are successfully and uniformly doped into MoS_2_. The composite is composed of Fe, Mo, S, C, and O.

**Figure 2:**
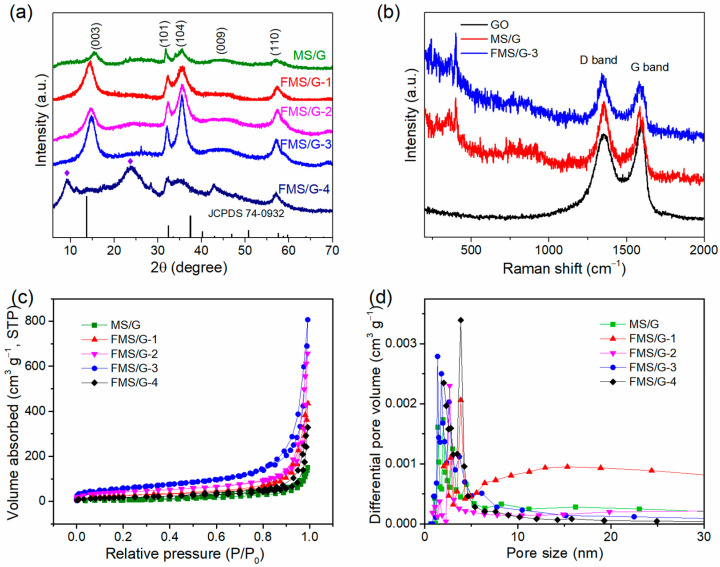
(**a**) XRD pattern of MS/G, FMS/G-1, FMS/G-2, FMS/G-3, and FMS/G-4 composite, (**b**) Raman spectra, (**c**) N_2_ sorption isotherms, (**d**) BJH pore size distribution plot of MS/G, FMS/G-1, FMS/G-2, FMS/G-3, and FMS/G-4 composite.

To investigate the crystal structure, an XRD pattern analysis was performed, as illustrated in Figure 2a. All the samples, MS/G, FMS/G-1, FMS/G-2, FMS/G-3, and FMS/G-4, revealed a broad diffraction peak at 2θ = 25.8°, representing the graphene’s characteristic peak, suggesting that graphene oxide was efficiently reduced during the production process. The other diffraction peaks in the MS/G composite correspond to the (003), (101), (104), (009), and (110) planes of the standard MoS_2_ structure (JCPDS 74-0932) [24]. For FMS/G-1, FMS/G-2, FMS/G-3, and FMS/G-4, the diffraction peaks in each composite shift slightly toward higher angles compared to undoped MS/G. Additionally, no extra signals for the iron phase are detected in the patterns of FMS/G-1, FMS/G-2, and FMS/G-3, suggesting that Fe is effectively doped into the MoS_2_ lattice [29]. Furthermore, the additional diffraction peaks (~7.8°) are present in the FMS/G-4 composite, attributed to Fe_3_S_4_ (JCPDS 89-2000) [30]. This indicates that when the dopant amount reaches 0.4 mmol, iron atoms cannot be fully incorporated into the MoS_2_ structure, resulting in the coexistence of two phases in the sample.

Raman spectroscopy was performed on FMS/G-3 and undoped MS/G to analyze their structural features. As depicted in Figure 2b, both samples exhibited the characteristic D band at 1340 cm^−1^, indicating defects and disorder, and the G band at 1575 cm^−1^, corresponding to the stretching vibrations associated with the sp^2^-hybridized carbon atoms [31]. Both FMS/G-3 and undoped MS/G exhibit higher I_D_/I_G_ ratios compared to graphene oxide (GO), indicating that GO has been successfully reduced to reduced graphene oxide (rGO) during the hydrothermal reaction [32]. High temperature and pressure during the hydrothermal process facilitate water-mediated hydrogenation, converting oxidized carbon sites to C-H bonds while restoring sp^2^ hybridization and conjugated π-systems. Structural rearrangement reduces interlayer spacing, forming more ordered graphitic domains in rGO [32]. This reduction confirms the presence of sp^2^-hybridized carbons, which contribute to favorable electrochemical properties. Additional peaks observed in the 200–600 cm^−1^ range for both FMS/G-3 and undoped MS/G are attributed to the characteristic vibrations of MoS_2_. In particular, the peaks observed at 376 and 403 cm^−1^ are assigned to the E^1^_2g_ and A_1g_ modes of MoS_2_ [33].

The pore size distribution and SSA of MS/G, FMS/G-1, FMS/G-2, FMS/G-3, and FMS/G-4 are illustrated in Figure 2c. At relative pressures of 0.8–1.0, all the samples displayed a type IV Langmuir isotherm with a hysteresis loop, indicating the mesoporous nature of the samples [34]. The FMS/G-3 composite demonstrated a markedly higher SSA of 181 m^2^ g^−1^ compared to other samples, such as 106 m^2^ g^−1^ for MS/G, 136 m^2^ g^−1^ for FMS/G-1, 158 m^2^ g^−1^ for FMS/G-2, and 132 m^2^ g^−1^ for FMS/G-4. The SSA values increased significantly after iron doped into the MS/G, together with its mesoporous architecture, facilitates faster ion diffusion and more efficient electron transport. Figure 2d displays the pore size distributions of MS/G, FMS/G-1, FMS/G-2, FMS/G-3, and FMS/G-4, as determined by Barrett–Joyner–Halenda (BJH) analysis. The results reveal that pore sizes of the samples were between 1.5 and 4 nm, confirming their mesoporous nanostructures. Both the large SSA and mesoporous architecture contribute to improving the capacitive performance of the electrode materials.

**Figure 3:**
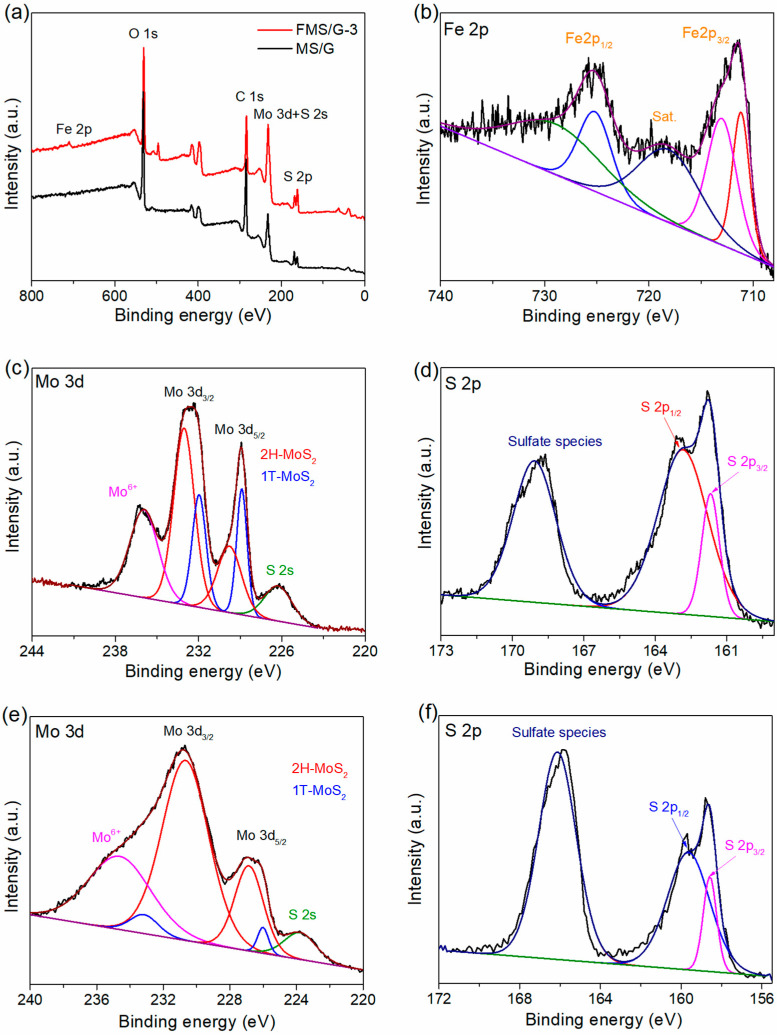
(**a**) XPS survey of MS/G and FMS/G-3, (**b**) Fe 2p spectrum, (**c**) Mo 3d spectrum, and (**d**) S 2p spectrum of FMS/G-3, (**e**) Mo 3d spectrum, and (**f**) S 2p spectrum of MS/G.

The chemical composition of the FMS/G-3 and MS/G composites were examined using XPS analysis. The survey spectrum in Figure 3a shows Fe, Mo, S, C, and O in the FMS/G-3, indicating that the composite was successfully synthesized. And the Mo, S, C, and O were clearly observed in the undoped MS/G composite. Detailed Fe 2p spectrum analysis in Figure 3b indicates the peaks for Fe 2p_3/2_ and Fe 2p_1/2_ at 711.6 and 725 eV, respectively, with each spin–orbit component further splitting into two distinct signals corresponding to Fe^2+^ and Fe^3+^ [35]. This splitting pattern is unique to materials containing both Fe^2+^ and Fe^3+^ [36]. The Mo 3d spectrum of FMS/G-3 in Figure 3c displays four distinct peaks corresponding to Mo^6+^, Mo 3d_3/2_, Mo 3d_5/2_, and S 2s [37]. The peak at 236.6 eV was ascribed to Mo^6+^. The main peaks at 232.6 and 229.1 eV were respectively ascribed to Mo 3d_3/2_ and Mo 3d_5/2_, which are characteristic of MoS_2_, while the S 2s orbital of MoS_2_ appeared with a representative peak at 226.7 eV. The S 2p spectrum (Figure 3d) shows two doublets at 161.6 eV (S 2p_3/2_) and 162.8 eV (S 2p_1/2_), with a new peak at 169.0 eV assigned to sulfate species [38]. The undoped MS/G shows the typical Mo 3d_3/2_ and Mo 3d_5/2_ of 2H phase at 233.1 and 226.85 eV, and the Mo 3d_3/2_ and Mo 3d_5/2_ peaks of 1T phase appeared at 230.7 and 226.03 eV. Hence, the peaks of Mo 3d_3/2_ and Mo 3d_5/2_ in FMS/G-3 shifted to higher binding energy compared to the undoped MS/G. This indicated that the Fe ions were uniformly doped into the MoS_2_ substrate [17]. The XPS analysis of FMS/G-1, FMS/G-2, and FMS/G-4 composites were shown in Figure S1. All the binding energies and peak positions corresponding to Mo of MS/G, FMS/G-1, FMS/G-2, FMS/G-3, and FMS/G-4 composites are listed in Table 1. It displays the peaks of Mo shifting toward higher values as the ratio of Fe/Mo gradually increases to 0.3. The C 1s spectrum in Figure S2 shows peaks at 284.8, 285.3, and 288.0 eV, corresponding to C=C, C-O, and C=O bonds, respectively [39]. These findings confirm the successful synthesis of the FMS/G composite and demonstrate that iron atoms are effectively incorporated into the MoS_2_ crystal lattice.

**Figure 4:**
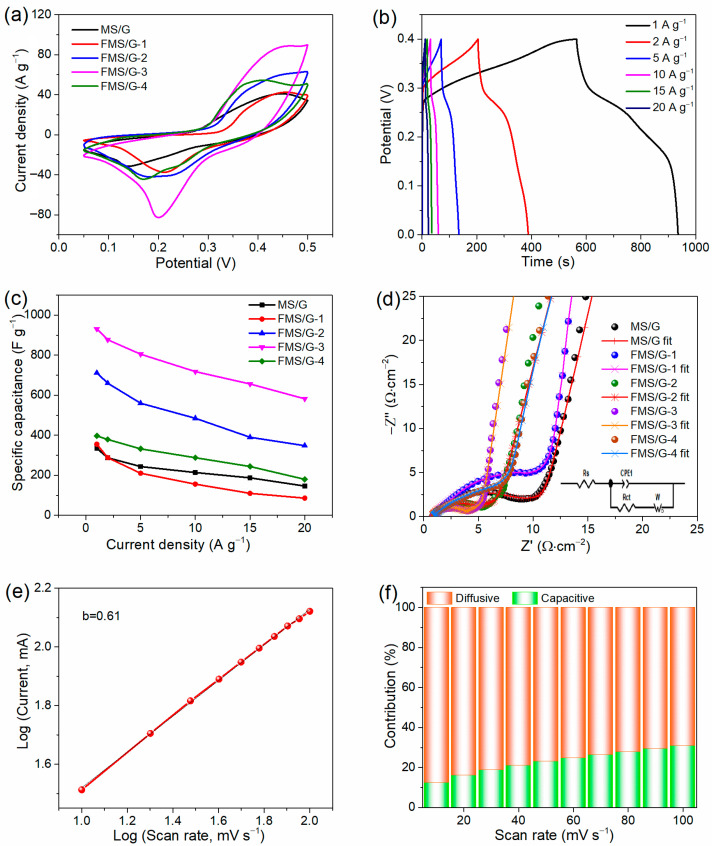
(**a**) CV curves of MS/G, FMS/G-1, FMS/G-2, FMS/G-3, and FMS/G-4 composites at 50 mV s^−1^, (**b**) GCD curves at different current densities of FMS/G-3 composite, (**c**) specific capacitance, (**d**) Nyquist plots of MS/G, FMS/G-1, FMS/G-2, FMS/G-3, and FMS/G-4 composites, (**e**) linear fitting plots of log (*i*) vs. log (*ν*), (**f**) capacitive contribution of FMS/G-3 composite.

The electrochemical performance of the FMS/G-3 composite as a positive electrode for SCs was assessed in a 3 M KOH electrolyte using a three-electrode configuration. CV curves of MS/G, FMS/G-1, FMS/G-2, FMS/G-3, and FMS/G-4 recorded at 50 mV s^−1^ in the potential range between 0.05 and 0.5 V are displayed in Figure 4a. Distinct redox peaks were observed for all samples, confirming their battery-type behavior. Notably, the FMS/G-3 composite shows the largest CV integral area compared to MS/G, FMS/G-1, FMS/G-2, and FMS/G-4, suggesting superior electrochemical performance. Additionally, the CV area of iron-doped MoS_2_/G composites is larger than that of undoped MoS_2_/G, attributed to the multiple oxidation states of iron and molybdenum, which facilitate more redox reactions [40]. Figure S3 shows the CV profiles of the FMS/G-3 composite at scan rates from 10 to 100 mV s^−1^, all of which reveal clear redox peaks, confirming its battery-type characteristics. According to the results of Figure S3, the specific capacitances of the FMS/G-3 composite at various scan rates were calculated as shown in Figure S4. The highest specific capacitance was 777 F g^−1^ at the scan rate of 10 mV s^−1^. Even at 100 mV s^−1^, the specific capacitance remains at 321 F g^−1^. To gain further insight, GCD tests were carried out, and the curves are shown in Figure 4b, acquired with current densities ranging from 1 to 20 A g^−1^. It exhibited nonlinear profiles, indicative of battery-type capacitance and consistent with the CV observations. The corresponding specific capacitances, calculated from these GCD curves, are summarized in Figure 4c. The FMS/G-3 composite delivered outstanding respective values of 931, 877, 805, 718, 656, and 582 F g^−1^ at 1, 2, 5, 10, 15, and 20 A g^−1^, outperforming all other samples. Even at 20 A g^−1^, it maintained 63% of its initial capacitance, confirming excellent rate performance. At 1 A g^−1^, increasing the iron content from 0 to 0.4 mmol enhanced the specific capacitance from 335 to 931 F g^−1^; however, exceeding 0.3 mmol results in a decline in capacitance. This behavior aligns with the EIS profiles in Figure 4d, where the high-frequency semicircle represents charge–transfer resistance (Rct), whereas the low-frequency linear region reflects ion transport in the electrolyte. The fitting parameters from the EIS plots of MS/G, FMS/G-1, FMS/G-2, FMS/G-3, and FMS/G-4 composites are depicted in Table S1. The FMS/G-3 composite demonstrated a lower R_ct_ value of 2.86 Ω·cm^−2^ compared to MS/G (8.12 Ω·cm^−2^), FMS/G-1 (3.94 Ω·cm^−2^), FMS/G-2 (3.92 Ω·cm^−2^), and FMS/G-4 (7.83 Ω·cm^−2^). FMS/G-3 has the lowest *W*-*R* (4.848 Ω·cm^2^) and highest *W*-*T* (1.232 s^1/2^), indicating the fastest diffusion [41]. Figure S5 shows the cycling stability of the FMS/G-3 composite tested over 10,000 charge–discharge cycles at 10 A g^−1^. After cycling, the electrode retained 90.5% of its capacitance, a considerable improvement compared to the undoped MS/G composite. This enhancement is mainly due to iron incorporation, which enlarges the surface area and accelerates ion transport. The remarkable electrochemical performance of FMS/G-3 can be ascribed to several factors: 1. Iron incorporation creates lattice vacancies in MoS_2_, enabling more redox-active sites and improving charge storage capability. 2. Iron doping prevents agglomeration of MoS_2_-active sites on graphene sheets, ensuring uniform anchoring and increased effective surface area. This shortens ion diffusion and electron transfer pathways, improving capacitance and rate capability. 3. The highly conductive graphene matrix protects FMS-active sites from degradation during prolonged charge–discharge cycles, ensuring outstanding cycling stability. The energy storage mechanism of the FMS/G-3 electrode was examined by applying the following equations to the analysis of CV curves [42]:

(1) $$i=a\nu^{b}$$

(2) $$i\left(V\right)=k_{1}\nu +k_{2}\nu^{\frac{1}{2}}$$

Here, *k*_1_, *k*_2_, *a*, and *b* denote the constants, *ν* represents the scan rate, and *i* indicates the current. A *b*-value between 0.5 and 1 indicates a combined contribution from capacitive- and diffusion-controlled charge storage mechanisms. Figure 4e demonstrates that the FMS/G-3 electrode achieved a *b*-value of 0.61, reflecting the coexistence of capacitive- and diffusion-controlled charge storage. Meanwhile, Figure 4f shows that with increasing scan rates, the capacitive contribution became more pronounced due to the restricted ion diffusion at higher sweep rates. Moreover, as indicated in Table 2, the FMS/G-3 composite as a positive electrode exhibits superior performance in comparison to the electrode materials for supercapacitors that have been recently reported.

**Figure 5:**
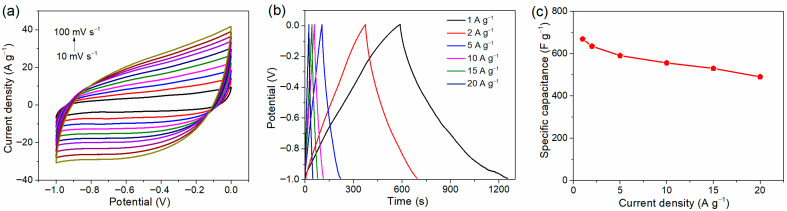
(**a**) CV curves at various scan rates, (**b**) GCD curves at different current densities, and (**c**) specific capacitance of FMS/G-3 composite at negative potential window.

The FMS/G-3 composite also demonstrated excellent performance as a negative electrode in Figure 5. The CV curves in Figure 5a performed at scan rates between 10 and 100 mV s^−1^ and maintained a quasi-rectangular profile even under high scan rates, confirming its superior capacitive behavior. To learn more about the FMS/G-3 composite’s electrochemical performance, GCD measurements were carried out with current densities varying from 1 to 20 A g^−1^ in Figure 5b. It displays well-defined triangular shapes, confirming excellent capacitive characteristics in agreement with the CV results. Figure 5c shows the specific capacitance of the FMS/G-3 composite, derived from GCD measurements, with excellent values of 669, 635, 591, 556, 530, and 490 F g^−1^ at 1, 2, 5, 10, 15, and 20 A g^−1^, respectively. The performance of the FMS/G-3 composite as a negative electrode was compared to the reported works as shown in Table 3.

**Figure 6:**
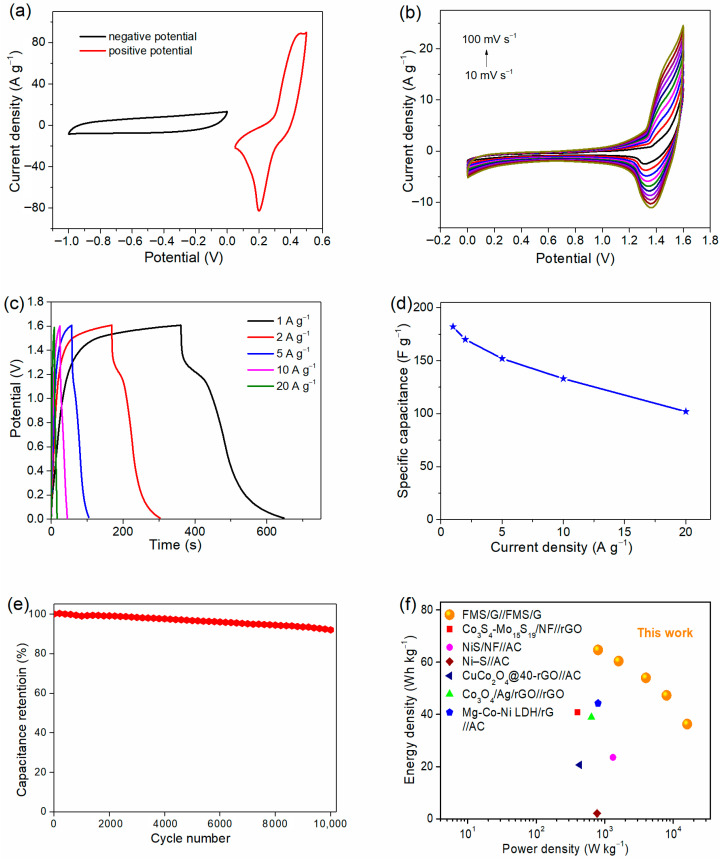
(**a**) CV curves of FMS/G-3 as positive and negative electrode at 50 mV s^−1^, (**b**) CVs at various sweep rates (10–100 mV s^−1^), (**c**) GCD curves at various current densities (1–20 A g^−1^), (**d**) specific capacitance vs. current density, (**e**) cycling stabilities for 10,000 cycles, and (**f**) Ragone plot of the FMS/G-3//FMS/G-3 SC device.

A SC device was constructed using FMS/G-3 as both positive and negative electrodes to assess the practical performance. The CV curves of FMS/G-3 electrodes at 50 mV s^−1^ at different voltage windows are shown in Figure 6a. Within the range of −1 to 0 V, the electrode exhibited capacitive features, while from 0.05 to 0.5 V, distinct faradaic behavior was observed. To identify a suitable operating window for the FMS/G-3//FMS/G-3 device, CV and GCD measurements were conducted over multiple potential ranges, as shown in Figure S6a,b. The potential range of 0 to 1.6 V was identified as stable for device operation, with noticeable polarization occurring at 1.8 V due to the oxygen evolution reaction. Therefore, 0 to 1.6 V was selected as the optimal potential window, and CV plots of the device at different scan rates are shown in Figure 6b, displaying redox behavior while maintaining curve shapes, indicating rapid electron transfer and excellent charge–discharge capabilities. The GCD curves of the SC device at various current densities are demonstrated in Figure 6c, with capacitance values derived from these profiles as shown in Figure 6d. The device exhibited 182 F g^−1^ at 1 A g^−1^ and sustained 102 F g^−1^ at 20 A g^−1^. The cycling stability of the SC device was examined at 10 A g^−1^ over 10,000 GCD cycles in Figure 6e, where it retained 92% of its capacitance, confirming excellent long-term durability. This superior electrochemical behavior resulted from the outstanding characteristics of FMS/G-3 when used as both anode and cathode. The Ragone plot of the FMS/G-3//FMS/G-3 device is presented in Figure 6f, demonstrating an impressive energy density of 64.7 Wh kg^−1^ at a power density of 800 W kg^−1^. The energy density remains at 36.3 Wh kg^−1^ even at a high-power density of 16,015 W kg^−1^, outperforming many previously documented SC devices such as Co_3_S_4_-Mo_15_S_19_/NF//rGO (40.8 W h kg^−1^ at 400 W kg^−1^) [59], NiS/NF//AC (23.58 W h kg^−1^ at 1320 W kg^−1^) [43], Ni-S//AC (2.142 W h kg^−1^ at 775 W kg^−1^) [44], CuCo_2_O_4_@40-rGO//AC (38.9 Wh kg^−1^ at 640 W kg^−1^) [60], Co_3_O_4_/Ag/rGO//rGO (35 W h kg^−1^ at 801 W kg^−1^) [61], and Mg-Co-Ni LDH/rG//AC (44.3 W h kg^−1^ at 800 W kg^−1^) [62].

## 3. Experimental

### 3.1. Materials

Ferric chloride hexahydrate (FeCl_3_·6H_2_O), thioacetamide, sodium dithiomolybdate (Na_2_MoO_4_·2H_2_O), potassium hydroxide (KOH), Poly(vinylidene fluoride) (PVDF), *N*-methyl-2-pyrrolidinone, ethylene glycol (EG), and graphene oxide (GO) powder were obtained from Aladdin Ltd. of Shanghai, China. All chemicals were utilized without any further purification.

### 3.2. Synthesis of FMS/G Composite

Take 80 mg of graphene oxide (GO), grind it into a powder, and dissolve it in 20 mL of deionized water, followed by ultrasonic treatment for 40 min. Dissolve 0.3 mmol of iron(III) chloride, 1.5 mmol of sodium molybdate, and 3 mmol of thioacetamide in 10 mL of deionized water. Add 0.5 mL of acetic acid to the aforementioned mixture and subject it to ultrasonic treatment for 30 min time duration to ensure thorough mixing. The solution was then transferred into a 50 mL hydrothermal reactor, which was then sealed and heated for 12 h time duration in an oven at 180 °C. Upon completion, the reaction mixture was brought to ambient temperature, after which the product (solid) was isolated and repeatedly rinsed with deionized water and ethanol to ensure thorough purification. Finally, place the processed material in an oven and dry it thoroughly at 70 °C. Grind the dried sample into a powder to obtain iron-doped molybdenum sulfide/graphene composite material powder, labeled as FMS/G-3.

To investigate the impact of different doping amounts of Fe on the composite material, composite materials were prepared with molar ratios of iron(III) chloride to sodium molybdate (Fe/Mo) set at 0.1:1.5, 0.2:1.5, and 0.4:1.5, and labeled as FMS/G-1, FMS/G-2, and FMS/G-4, respectively. An undoped molybdenum sulfide/graphene composite material (MS/G) was also prepared using the aforementioned method for comparative analysis.

### 3.3. Material Characterization

The morphological characteristics of the synthesized materials were characterized using a Hitachi JSM-7900F field emission scanning electron microscope (FE-SEM) from Hitachi (Tokyo, Japan) and a Tecnai G2 F20/F30 transmission electron microscope (TEM) from FEI Company (Hillsboro, OR, USA). Elemental composition and distribution were analyzed via energy-dispersive X-ray spectroscopy (EDS) from Hitachi (Tokyo, Japan). Powder X-ray diffraction (XRD) patterns were obtained using a Rigaku Miniflex600 diffractometer (Rigaku, Akishima, Japan) at 5°·min^−1^, whereas a LabRAM HR800 Raman spectrometer (Horiba Jobin Yvon, Longjumeau, France) was used to record Raman spectra. Specific surface area (SSA) and pore size distribution determination were performed using an SSA-4300 analyzer from Builder (Beijing, China). Furthermore, X-ray photoelectron spectroscopy (XPS; Thermo ESCALAB 250XI, Waltham, MA, USA) with an Al Kα monochromatic source was employed for characterizing the chemical states of the composite.

### 3.4. Electrochemical Analysis

The sample’s electrochemical performance was evaluated using a standard three-electrode setup. The preparation of the working electrode includes the slurry formation by mixing the sample with polyvinylidene fluoride (PVDF) and carbon black in an 8:1:1 ratio, using *N*-methyl-2-pyrrolidinone as the solvent. After ultrasonic treatment, the slurry was applied onto a nickel foam piece (1 cm × 1 cm), which was then oven-dried before use. For the electrochemical setup, a platinum foil was used and served as the auxiliary electrode, while Ag/AgCl was the reference, and the FMS/G composite was tested at a mass of 4 mg.

### 3.5. Fabrication of the Supercapacitor Device

SC device construction was carried out using the FMS/G composite serving as both the positive as well as negative electrodes, with a 3 M KOH solution as the electrolyte. The respective positive and negative electrode’s mass ratios were determined to be 7 mg and 4 mg.

The specific capacitance (*C*) of the electrodes was calculated from the charge–discharge curves using Equation (3):

(3) $$C=\frac{I\Delta t}{m\Delta V}$$

where *I* is the discharge current (A), *m* is the active material’s mass loading (g), Δt is the discharge time, and Δ*V* is the applied potential window.

The energy density (*E*, in Wh kg^−1^) and power density (*P*, in W kg^−1^) of the SC device were calculated using Equations (4) and (5):

(4) $$E=\frac{C_{cell}\;\Delta V^{2}}{2\times 3.6}\;$$

(5) $$P=\frac{E3600}{t_{discharge}}\;$$

where Δ*V* is the applied voltage range, *C_cell_* is the specific capacitance of the SC device, and *t_discharge_* is the discharge time during the galvanostatic charge–discharge (GCD) test.

## 4. Conclusions

In summary, we successfully synthesized the FMS/G-3 composite, which serves as both a highly effective positive and negative electrode material for SC devices. This material holds promise for scalable production and can be adapted to create other nanostructure-based hybrid architectures. The inclusion of iron in the composite introduces numerous active sites, significantly enhancing its electrochemical properties. The FMS/G-3 composite exhibited a remarkably high SSA of 181 m^2^ g^−1^ and delivered a maximum 931 F g^−1^ specific capacitance at 1 A g^−1^. In addition, the electrode showed outstanding durability, maintaining 90.5% of its capacitance following 10,000 cycles. The FMS/G-3//FMS/G-3 SC device exhibits excellent performance, achieving an outstanding energy density of 64.7 Wh kg^−1^ at a power density of 0.8 kW kg^−1^. Notably, it still maintained a considerable 36.3 Wh kg^−1^ even with a 16.02 kW kg^−1^. Additionally, the device shows exceptional durability, maintaining 92% of its capacitance after 10,000 cycles. The unique structure resulting from the incorporation of iron into MoS_2_ nanoflowers represents a significant advancement with promising applications, particularly in energy conversion and storage devices for modern electronics. Furthermore, this work presents a novel strategy for designing electrode materials for efficient application in energy storage systems, demonstrating significant promise for real-world applications.

## Figures and Tables

**Table 1:** Variation peaks of the deconvoluted Mo with different iron doping.

Samples	Mo 3d_3/2_ (eV)		Mo 3d_5/2_ (eV)	
	2H	1T	2H	1T
MS/G	233.05	230.7	226.8	226.0
FMS/G-1	232.7	231.4	229.2	228.2
FMS/G-2	232.9	231.7	229.8	228.6
FMS/G-3	233.1	231.9	229.8	228.8
FMS/G-4	233.5		232.2	230.4

**Table 2:** Comparison of the electrochemical performance of the reported materials as positive electrode for supercapacitors.

Materials	Specific Capacitance	Electrolyte	Stability	Ref.
NiS	613 F g^−1^ at 1.1 A g^−1^	3 M KOH	97% (5000 cycles)	[43]
Zn-NiS/G	442.66 F g^−1^ at 1 A g^−1^	6 M KOH	-	[44]
Ni-Cr_2_S_3_	187.53 F g^−1^ at 0.5 A g^−1^	3 M KOH	93.31% (5000 cycles)	[45]
SS-NiS@3DNF	470 F g^−1^ at 1 A g^−1^	2 M KOH	88% (6700 cycles)	[46]
CuS/CdS	543.6 F g^−1^ at 1 A g^−1^	3 M KOH	89.06% (10,000 cycles)	[47]
CuCo_2_S_4_	831.7 F g^−1^ at 20 mV s^−1^	3 M KOH	78.8% (10,000 cycles)	[48]
WS_2_@VS_2_	615 F g^−1^ at 2.5 A g^−1^	1 M KOH	98.85% (5000 cycles)	[49]
Zn_0.7_Fe_0.3_S	700 F g^−1^ at 0.5 A g^−1^	6 M KOH	80% (2000 cycles)	[50]
FMS/G	931 F·g^−1^ at 1 A·g^−1^	3 M KOH	90.5% (10,000 cycles)	This work

**Table 3:** Comparison of the electrochemical performance of the reported materials as negative electrode for supercapacitors.

Materials	Specific Capacitance	Electrolyte	Ref.
NSA-FeS_2_/polyaniline	976 F g^−1^ at 0.5 A g^−1^	1 M Na_2_SO_3_	[51]
Fe_3_Mo_3_C/Mo_2_C@CNTs	202.3 F g^−1^ at 1 A g^−1^	1 M KOH	[52]
Mo_2_C/Fe_3_Mo_3_C	354.9 F g^−1^ at 1 A g^−1^	1 M KOH	[53]
SnO_2_	325 F g^−1^ at 1 A g^−1^	2 M Na_2_SO_4_	[54]
Bi_2_S_3_	565 F g^−1^ at 1 A g^−1^	2 M Na_2_SO_4_	[55]
MoO_2_	411 F g^−1^ at 1 A g^−1^	1 M Na_2_SO_4_	[56]
GO/Fe-VO_4_-BIPY	190 F g^−1^ at 0.5 A g^−1^	6 M KOH	[57]
(Fe/Bi)_2_S_3_	610 F g^−1^ at 1 A g^−1^	6 M KOH	[58]
FMS/G	669 F·g^−1^ at 1 A·g^−1^	3 M KOH	This work

## Data Availability

The data presented in this study are available upon request from the corresponding author.

## Supplementary Materials

The following supporting information can be downloaded at: https://www.mdpi.com/article/10.3390/molecules30204045/s1, Figure S1: Survey and Mo 3d spectrum of FMS/G-1, FMS/G-2 and FMS/G-4 composites; Figure S2: C 1s spectrum of FMS/G-3; Figure S3: CV curves at various scan rates of FMS/G-3 composite at positive potential window; Figure S4: Specific capacitance of FMS/G-3 calculated by CV curves; Figure S5: Cycling stabilities for 10,000 cycles of FMS/G-3 composite; Figure S6: CV curves and GCD curves at different potential ranges of device. Table S1: Fitting parameters for MS/G, FMS/G-1, FMS/G-2, FMS/G-3, and FMS/G-4 composites.
