# Supplementary material for: Iron-Doped Molybdenum Sulfide Nanoflowers on Graphene for High-Performance Supercapacitors
Source: Molecules. 2025 Oct 10;30(20):4045. doi: 10.3390/molecules30204045 (PMC12566374; doi:10.3390/molecules30204045)
Supplement: Supplementary file 1 [file molecules-30-04045-s001.zip › molecules-3876388-supplementary.pdf]

Supplementary Materials

Iron-Doped Molybdenum Sulfide Nanoflowers on Graphene for High-Performance Supercapacitors

Xuyang Li [1,*], Mingjian Zhao [2], Shuyi Li [1], Shiyuan Cheng [1], Yiting Zuo [1], Kaixuan Wang [2] and Meng Guo [2,*]

[1] School of Chemistry and Pharmaceutical Engineering, Nanyang Normal University, Nanyang 473061, China
[2] School of Biological and Chemical Engineering, Nanyang Institute of Technology, Nanyang 473004, China
* Correspondence: 20181032@nynu.edu.cn (X.L.); 3132073@nyist.edu.cn (M.G.)

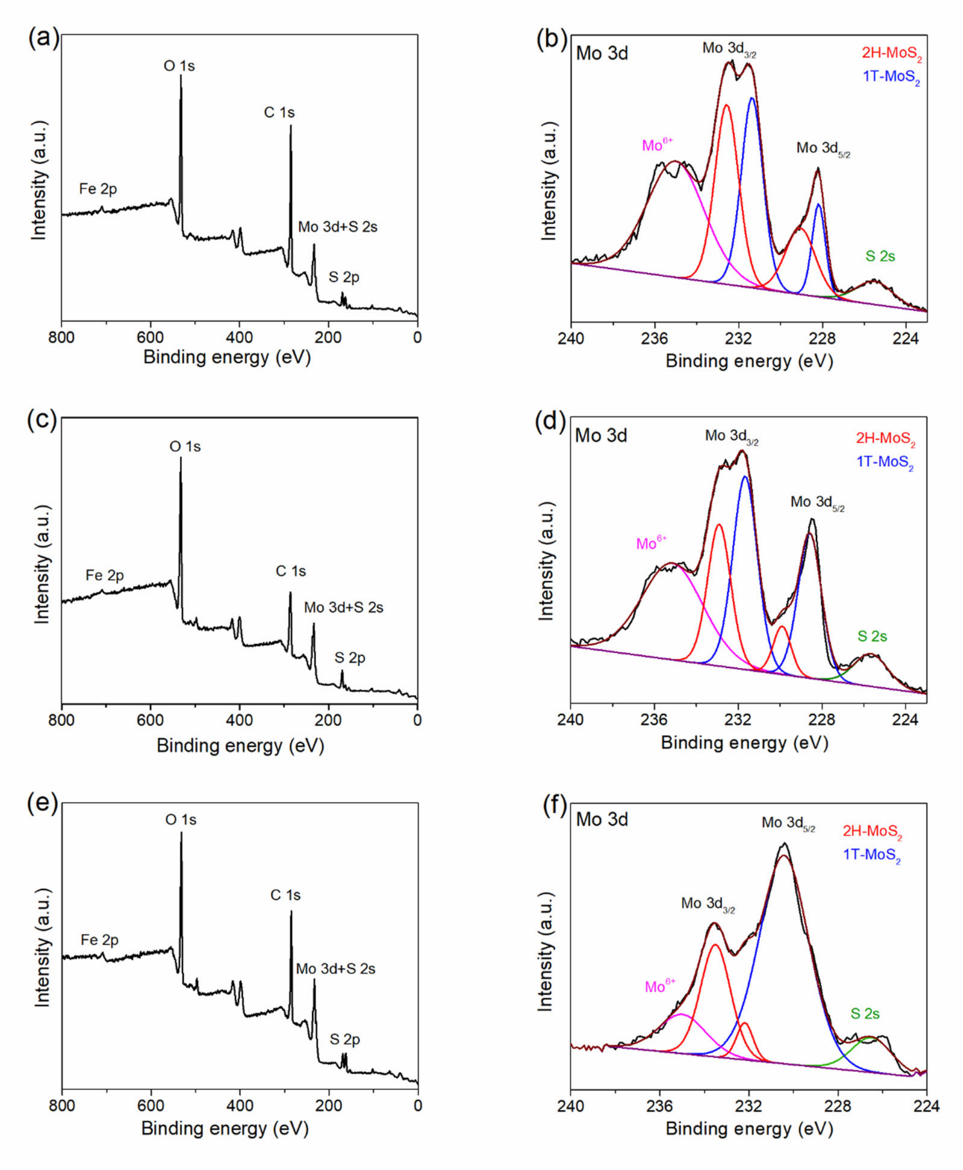

Figure S1. (a,c,e) Survey and (b, d,f) Mo 3d spectrum of FMS/G-1, FMS/G-2 and FMS/G-4 composites

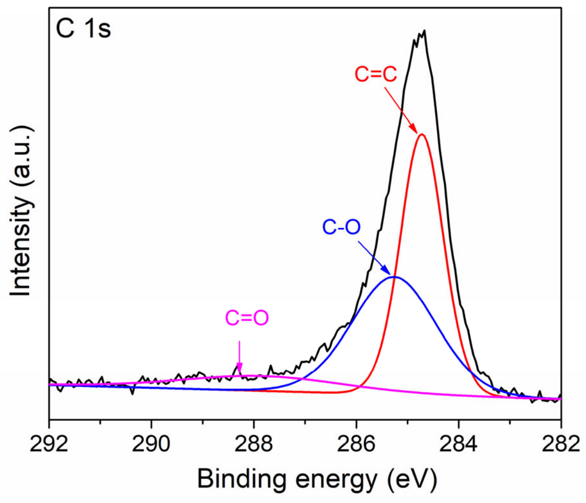

Figure S2. C 1s spectrum of FMS/G-3.

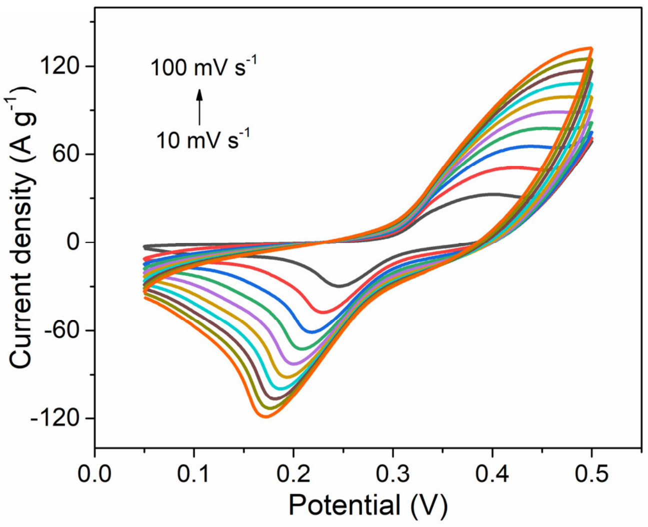

Figure S3. CV curves at various scan rates of FMS/G-3 composite at positive potential window

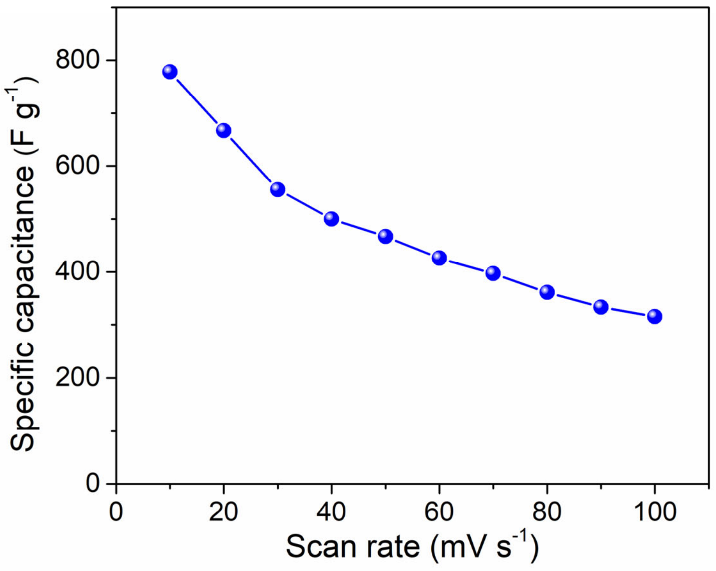

Figure S4. Specific capacitance of FMS/G-3 calculated by CV curves

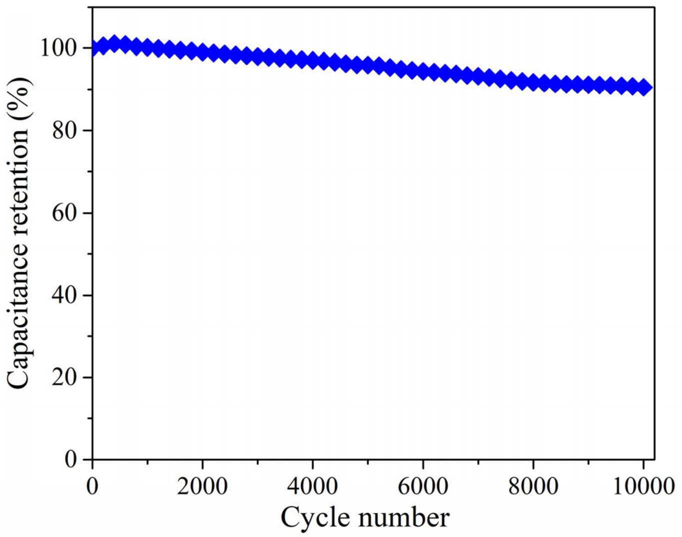

Figure S5. Cycling stabilities for 10000 cycles of FMS/G-3 composite

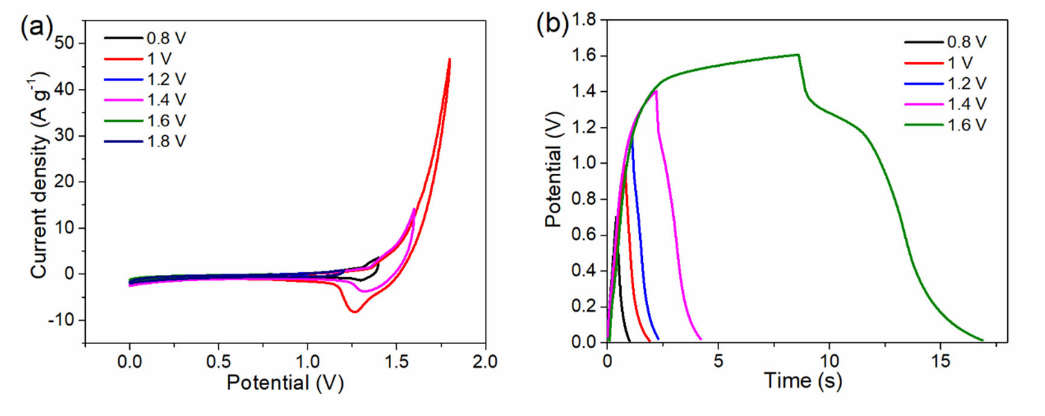

Figure S6. CV curves and GCD curves at different potential ranges of device

Table S1 Fitting parameters for MS/G, FMS/G-1, FMS/G-2, FMS/G-3, and FMS/G-4 composites

Samples	R_s	CPE1-T	CPE1-P	R_{ct}	W-R	W-T	W-P
MS/G	0.7732	0.0022346	0.70508	8.118	6.734	1.106	0.44325
FMS/G-1	0.79602	0.001864	0.76383	3.936	5.785	0.7689	0.4354
FMS /G-2	0.79572	0.0018565	0.76386	3.916	6.187	0.83805	0.43722
FMS /G-3	0.84769	0.0015642	0.74347	2.863	4.848	1.232	0.46322
FMS /G-4	0.91551	0.021429	0.62369	7.83	8.528	0.63145	0.48463
